# Arterial Oxygenation in Traumatic Brain Injury—Relation to Cerebral Energy Metabolism, Autoregulation, and Clinical Outcome

**DOI:** 10.1177/0885066620944097

**Published:** 2020-07-27

**Authors:** Teodor Svedung Wettervik, Henrik Engquist, Timothy Howells, Samuel Lenell, Elham Rostami, Lars Hillered, Per Enblad, Anders Lewén

**Affiliations:** 1 Department of Neuroscience, Section of Neurosurgery, 8097 Uppsala University, Uppsala, Sweden; 2 Department of Surgical Sciences/Anesthesia and Intensive Care, 8097 Uppsala University, Uppsala, Sweden

**Keywords:** autoregulation, energy metabolism, hyperoxia, neurointensive care, traumatic brain injury

## Abstract

**Background:**

Ischemic and hypoxic secondary brain insults are common and detrimental in traumatic brain injury (TBI). Treatment aims to maintain an adequate cerebral blood flow with sufficient arterial oxygen content. It has been suggested that arterial hyperoxia may be beneficial to the injured brain to compensate for cerebral ischemia, overcome diffusion barriers, and improve mitochondrial function. In this study, we investigated the relation between arterial oxygen levels and cerebral energy metabolism, pressure autoregulation, and clinical outcome.

**Methods:**

This retrospective study was based on 115 patients with severe TBI treated in the neurointensive care unit, Uppsala university hospital, Sweden, 2008 to 2018. Data from cerebral microdialysis (MD), arterial blood gases, hemodynamics, and intracranial pressure were analyzed the first 10 days post-injury. The first day post-injury was studied in particular.

**Results:**

Arterial oxygen levels were higher and with greater variability on the first day post-injury, whereas it was more stable the following 9 days. Normal-to-high mean pO_2_ was significantly associated with better pressure autoregulation/lower pressure reactivity index (*P* = .02) and lower cerebral MD-lactate (*P* = .04) on day 1. Patients with limited cerebral energy metabolic substrate supply (MD-pyruvate below 120 µM) and metabolic disturbances with MD-lactate-/pyruvate ratio (LPR) above 25 had significantly lower arterial oxygen levels than those with limited MD-pyruvate supply and normal MD-LPR (*P* = .001) this day. Arterial oxygenation was not associated with clinical outcome.

**Conclusions:**

Maintaining a pO_2_ above 12 kPa and higher may improve oxidative cerebral energy metabolism and pressure autoregulation, particularly in cases of limited energy substrate supply in the early phase of TBI. Evaluating the cerebral energy metabolic profile could yield a better patient selection for hyperoxic treatment in future trials.

## Introduction

Ischemic and hypoxic secondary injury events are common in traumatic brain injury (TBI).^1,2^ Maintaining an adequate cerebral blood flow (CBF) and delivery of oxygen is essential in TBI management.^3^ Brain tissue oxygenation (pBtO_2_) below 20 to 29 mm Hg is associated with poor clinical outcome.^4,5^ The pBtO_2_ correlates with regional CBF^6^ and arterial oxygen content.^7^ High intracranial pressure (ICP) and low cerebral perfusion pressure (CPP) are associated with low pBtO_2_, but most hypoxic insults occur in the absence of these predictors^4^ and are probably caused by microvascular thrombosis or diffusion barriers such as cerebral edema.^8,9^

Increasing the fraction of the inspired O_2_ (Fio_2_), for example, by normobaric hyperoxia (NBO), in order to increase arterial and brain oxygenation, has been suggested as treatment for cerebral hypoxia in TBI.^10^ The treatment may compensate for ischemic hypoxia, overcome cerebral diffusion barriers, and improve mitochondrial function, thereby improving oxidative cerebral energy metabolism and reducing secondary brain injuries.^2^ Normobaric hyperoxia may increase pBtO_2_,^11^ but the effect on cerebral energy metabolism is more controversial. Early cerebral microdialysis (MD) studies demonstrated decreased cerebral lactate following NBO treatment, but pyruvate also decreased and the lactate-/pyruvate-ratio (LPR) remained unchanged.^10,12–15^ However, other studies found that the hyperoxic effect depends on the current energy metabolic state, as hyperoxia was associated with improved cerebral metabolic rate of oxygen (CMRO_2_) in tissue at risk of ischemia^16^ and improved LPR in tissue with high cerebral lactate before treatment initiation.^17^ Outcome studies using NBO protocols have shown equivocal results.^18,19^ Spiotta et al found better clinical outcome for TBI patients treated with combined ICP-/pBtO_2_- versus ICP-oriented treatment alone,^18^ whereas Martini et al found no difference in outcome in a similar study.^19^ Thus, it is clear that hypoxia should be strictly avoided in TBI; however, the optimal (higher) pO_2_ level in certain situations needs to be studied further.

The link between CBF autoregulation and oxygenation also needs to be further explored in TBI patients. Cerebral blood flow is controlled by changes in arterial blood pressure, pCO_2_, pO_2_, and cerebral energy metabolism.^20–23^ Arterial hypoxia leads to increases in CBF by vasodilation, to maintain normal pBtO_2_.^24,25^ This specific capacity can be estimated as the tissue oxygenation response (TOR), that is, the difference in increased pBtO_2_ compared to the arterial blood flow after an increase in Fio_2_.^26^ Tissue oxygenation response is low if the cerebral oxygen autoregulation is intact, that is, an increase in arterial oxygenation generates only a modest increase in pBtO_2_ due to cerebral autoregulation. The cerebral oxygen autoregulatory status, as assessed using TOR, correlates with cerebral pressure autoregulation (PRx) and clinical outcome.^27^

In this study, we aimed to further elucidate the role of arterial oxygenation, incidence of hypoxia, and hyperoxia and the relation to cerebral energy metabolism, cerebrovascular reactivity, and clinical outcome in severe TBI. We hypothesized that arterial hypoxia would be disadvantageous and hyperoxia possibly beneficial in relation to these outcome measures.

## Materials and Methods

### Patients

Patients with severe TBI admitted to our neurointensive care (NIC) unit at the Department of Neurosurgery at the University Hospital in Uppsala, Sweden, 2008 to 2018 were eligible for this retrospective study. Of 1001 patients, those 115 patients with mechanical ventilation and monitoring of arterial blood pressure, ICP, arterial blood gas (ABG), and cerebral energy metabolism (using cerebral MD) that did not develop total brain infarction were included.

Patients were treated in accordance with our standardized ICP-oriented treatment protocol to avoid secondary insults.^28,29^ Treatment goals were ICP ≤ 20 mm Hg, CPP ≥ 60 mm Hg, systolic blood pressure > 100 mm Hg, central venous pressure 0 to 5 mm Hg, pO_2_ > 12 kPa, arterial glucose 5 to 10 mmol/L (mM), hemoglobin (Hb) > 100 g/L, electrolytes within normal ranges, normovolemia and body temperature <38 °C. Patients were initially mildly hyperventilated (4.0-4.5 kPa) and normoventilated as soon as ICP allowed.

### Data Acquisition and Analysis

Intracranial pressure was monitored with either an intraparenchymal sensor device (Codman ICP Micro-Sensor, Codman & Shurtleff) or an intraventricular catheter (HanniSet, Xtrans, Smith Medical GmbH) with the tip in the frontal horn. Arterial blood pressure was measured invasively in the radial artery at heart level. Arterial blood gas data were generally analyzed in samples taken through the radial arterial line every fourth hour, more often if needed. Arterial blood gas samples were analyzed on an ABL800 FLEX instrument (Radiometer), running automatic calibrations every fourth hour and internal quality control samples twice daily. In addition, an external quality control program with monthly control samples administered by the accredited clinical chemistry laboratory at Uppsala university hospital was used. The physiological calculations mentioned below were done in the Odin software.^30^ Pressure reactivity index, traditionally calculated as the 5-minute correlation of 10-second averages of ICP and MAP,^31^ was used in combination with a bandpass filter, limiting the analysis to oscillations with periods of 15 to 55 seconds (PRx55-15).^32,33^

For MD, we used the 71 High Cut-Off Brain Microdialysis Catheter with a membrane length of 10 mm and a membrane cutoff of 100 kDa (M Dialysis). The MD catheter was placed in normal-appearing brain tissue in the right frontal lobe, adjacent to the ICP monitor. The catheters were perfused with a microinjection pump (106 MD Pump, M Dialysis) at a rate of 0.3 µL/min using custom made sterile artificial cerebrospinal fluid containing—NaCl 147 mmol/L (mM), KCl 2.7 mM, CaCl_2_ 1.2 mM, MgCl_2_ 0.85 mM and 1.5% human albumin. Cerebral interstitial glucose, lactate, pyruvate, and urea were measured hourly, with the CMA 600 analyzer or the ISCUSflex Microdialysis Analyzer (M Dialysis). The cerebral MD-urea was monitored to validate catheter performance.^34^ The analyzers were automatically calibrated when started, as well as every sixth hour using standard calibration solutions from the manufacturer (M Dialysis). Quality controls were performed every weekday at 2 different concentrations for each substance (CMA 600 and ISCUSflex). Total imprecision coefficient of variation was <10% for all analytes. The correlation between CMA600 and ISCUSflex data was found to allow for direct comparisons of patient data.^35^

### Outcome

Clinical outcome was assessed at 6 months post-injury, by specially trained personnel with structured telephone interviews, using the Extended Glasgow Outcome Scale (GOS-E), containing 8 categories of global outcome, from death to upper good recovery.^36–38^

### Statistical Analysis

The temporal course of pO_2_, Fio_2_, and Hb was calculated for each patient the first 10 days post-injury. Similarly, the percentage (%) of ABGs below our oxygenation target (pO_2_ < 12 kPa), hyperoxia (pO_2_ > 20 kPa), and anemia (Hb < 100 g/L) was calculated. The calculation was based on the number of blood gases above/below those thresholds versus the total number of blood gases on the same day. Analysis of the first 10 days after injury (N = 115) showed that day 1 had the highest pO_2_, the greatest pO_2_-variability and the highest prevalence of anemia, and therefore we chose to focus on this dynamic day for further data analysis. The statistical analyses below were performed both with and without patients treated with decompressive craniectomy (DC) the first day post-injury.

Mean values were calculated for each patient the first day and the difference in mean values between day 2 and 1 (Δ(Day 2 − Day 1)) post-injury for pO_2_ (kPa), pO_2_ < 12 kPa (%),pO_2_ > 20 kPa (%),pCO_2_ (kPa), Fio_2_ (%), Hb (g/L), ICP (mm Hg), CPP (mm Hg), PRx55-15, cerebral MD-glucose (mM), MD-pyruvate (µM), MD-lactate (mM), and MD-LPR in the software Odin. The mean values for ICP, CPP, and PRx55-15 were calculated using minute-by-minute data. The PRx55-15 was calculated minute-by-minute over a 5-minute moving window centered on the minute. The ABG parameters were analyzed approximately every fourth hour. The mean values of cerebral MD-glucose, pyruvate, lactate, and LPR were based on hourly analyses. All data were transferred to SPSS version 25 (IBM Corp) for further statistical analysis. We chiefly focused on pO_2_ as a measure of arterial oxygenation as this mean value carries more information than the percentage of hyperoxia/hypoxia. The Spearman correlation test was used to investigate the association between pO_2_ the first day post-injury with ICP, CPP, PRx55-15, cerebral MD-glucose, MD-lactate, MD-pyruvate, MD-LPR, and GOS-E. Similar correlation analyses were done using the (Δ(Day 2 − Day 1) values of the same variables. Those with missing values were excluded from the analyses. Multiple linear regression analyses were performed with PRx55-15 as the dependent variable for the first day post-injury and included the explanatory variables age, ICP, CPP, pCO_2_, and pO_2_. Differences in pO_2_ were evaluated in 4 patient groups that had complete data day 1 on pO_2_, cerebral pyruvate, and LPR (51 patients) in relation to their cerebral energy metabolic pattern (A) “*Energy metabolic disturbances, limited substrate supply*” (cerebral MD-LPR > 25 and MD-pyruvate < 120 µM, N = 4), (B) “*Normal energy metabolism, limited substrate supply*” (cerebral MD-LPR < 25 and MD-pyruvate < 120 µM, N = 21), (C) “*Energy metabolic disturbances, normal substrate supply*” (cerebral MD-LPR > 25 and MD-pyruvate > 120 µM, N = 6), and (D) “*Normal energy metabolism, normal substrate supply*” (cerebral MD-LPR < 25 and MD-pyruvate > 120 µM, N = 20). Group A versus B and C versus D were compared, respectively, with Student *t* tests. The cerebral MD-LPR threshold at 25 for metabolic disturbances was chosen in accordance with the consensus statement 2014.^39^ The cerebral pyruvate threshold for *limited* energy substrate supply at 120 µM was chosen based on previous studies suggesting that this is the highest pyruvate value for ischemic and the lowest value for nonischemic cerebral conditions.^40,41^ A *P* value <.05 was considered statistically significant.

### Ethics

All procedures performed in the studies involving humans were in accordance with the ethical standards of the institutional and national research committee and with the 1964 Helsinki declaration and its later amendments. Written informed consent was obtained from all individual patients included in the study or their next of kin.

## Results

### Demographic and Physiological Data

Of the 115 TBI patients included in the study, the mean age was 43 (±20) years and 76% were male. Median Glasgow Coma Scale Motor (GCS M) score was 5 (interquartile range [IQR]: 4-5). Twenty-one percent had a pupillary abnormality (anisocoria and/or fixed reaction) at admission. Seventy-five percent had diffuse injury (Marshall grade I-IV) and 25% focal brain injury (evacuated mass lesion V or nonevacuated mass lesion VI) on the first computed tomography scan. One percent of the patients had an impression fracture, 6% epidural hemorrhage (EDH), 20% acute subdural hemorrhage (ASDH), 6% traumatic subarachnoid hemorrhage (SAH), 35% cerebral contusions, 16% diffuse axonal injuries (DAIs), and 16% mixed intracranial injuries. Thirty-three percent had a thoracic injury and 41% had other extracranial injuries (abdominal, pelvic, spine, or extremities). Only one patient had extensive bleeding. Eleven percent was treated with thiopental and 15% with DC. Mean days in respirator was 12 (±8) days and mean stay at the NIC unit was 13 (±12) days. Outcome data 6 months after injury showed a mortality rate of 8% and median GOS-E was 5 (IQR: 3-7). The physiological data are presented in Table 1.

**Table 1.** Description of Arterial Blood Gases, Neurophysiological, and Neurochemical Parameters Day 1 and Δ(Day 2 − Day 1) Post-TBI.^a^

Physiological Variables	Day 1	Δ(Day 2 − Day 1)
PO_2_ (kPa)	18 (±4)	−2 (±4)
pO_2_ < 12 kPa (%)	7.9 (±15)	5 (±16)
pO_2_ > 20 kPa (%)	18 (±25)	−9 (±23)
SaO_2_ (%)	99 (±1)	0 (±1)
Fio_2_ (%)	38 (±7)	1 (±8)
P/F (pO_2_/Fio_2_)	49 (±15)	−5 (±10)
Hb (g/L)	113 (±17)	−4 (±11)
pCO_2_ (kPa)	4.7 (±0.5)	0.0 (±0.5)
ICP (mm Hg)	10 (±7)	2 (±4)
CPP (mm Hg)	76 (±11)	−1 (±8)
PRx55-15 (coefficient)	0.22 (±0.20)	−0.05 (±0.15)
Cerebral MD-glucose (mM)	2.6 (±1.3)	−0.2 (±1.0)
Cerebral MD-pyruvate (µM)	142 (±87)	4 (±67)
Cerebral MD-lactate (mM)	3.5 (±2.3)	0.1 (±1.2)
Cerebral MD-LPR	27 (±22)	−1 (±17)

Abbreviations: CPP, cerebral perfusion pressure; ICP, intracranial pressure; LPR, lactate-/pyruvate ratio; MD, microdialysis; PRx, pressure autoregulation index; SD, standard deviation; TBI, traumatic brain injury.

^a^ The values are given as mean (±SD) for all patients.

### Arterial Oxygenation and Its Relation to Extracranial Injuries and Clinical Outcome

**Figure 1.**
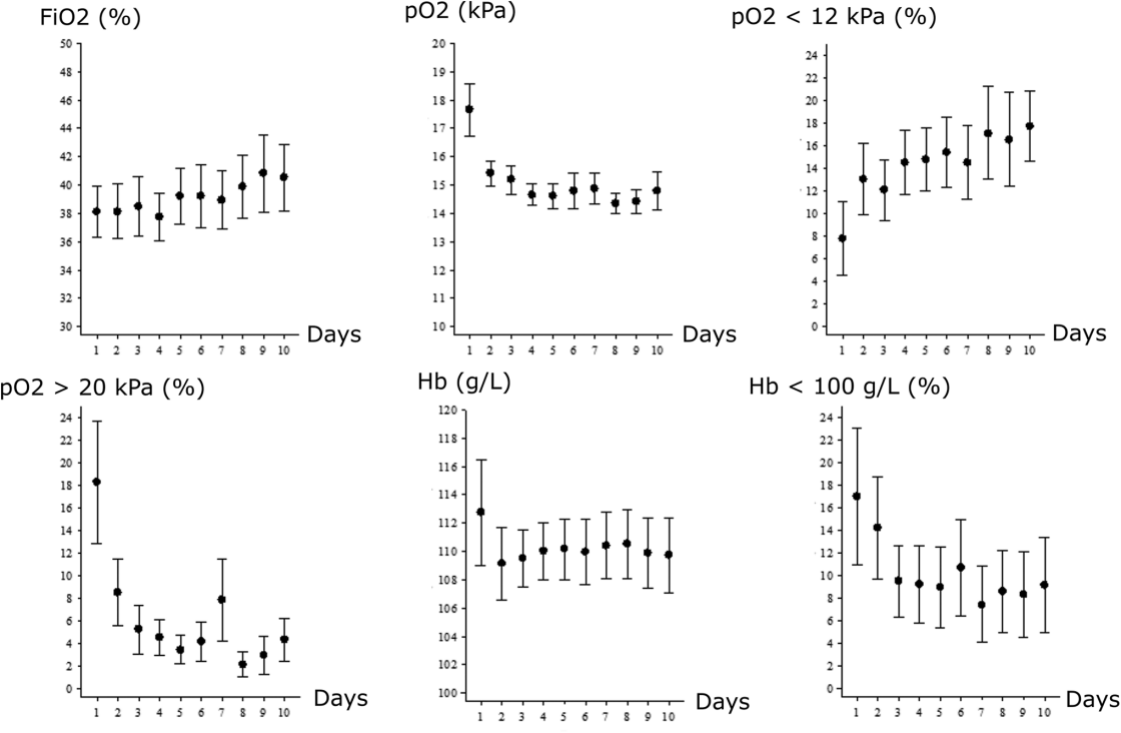
Arterial oxygenation—temporal course the first 10 days post-injury.

Mean arterial pO_2_ peaked the first day post-injury and gradually decreased (Figure 1). The percentage of ABGs with hyperoxia (pO_2_ > 20 kPa) decreased gradually, whereas the percentage of ABGs below our oxygen target (pO_2_ < 12 kPa) increased over the first 10 days. Fio_2_ gradually increased over the 10 days. The mean Hb was higher the first day and then slightly lower, but stable and above 100 g/L (Figure 1). The percentage of Hb <100 g/L was higher on day 1 and gradually decreased thereafter. There was no association between mean pO_2_, Hb, and Fio_2_ with thoracic or other extracranial injuries (data not shown). Mean pO_2_ events (% of ABGs) below 12 kPa or above 20 kPa on the first day post-injury did not correlate with clinical outcome (Table 2). Similarly, neither of these oxygen indices correlated with the length of stay at the NIC unit or number of days in respirator. Furthermore, there was still no correlation between mean pO_2_ and clinical outcome in any of the separate TBI subgroups based on injury type (impression fracture, EDH, ASDH, SAH, cerebral contusions, DAI, and mixed injuries, respectively).

**Table 2.** Arterial Oxygenation Day 1 Post-Injury—Relation to Intracranial Pressure Dynamics, Cerebral Energy Metabolism, and Clinical Outcome—Spearman Correlation Analyses.^a^

Physiological Variables	Mean pO_2_		pO_2_ < 12 kPa (%)		pO_2_ > 20 kPa (%)	
	r	*P* value	r	*P* value	r	*P* value
ICP	−0.14	.31	−0.06	.68	−*0.28*	***.04***
CPP	−0.04	.77	0.12	.37	0.07	.59
PRx55-15	−*0.30*	***.02***	0.24	.07	−0.20	.13
Cerebral MD-glucose	−0.06	.69	0.20	.15	−0.05	.71
Cerebral MD-pyruvate	−0.19	.17	*0.28*	***.046***	−0.08	.60
Cerebral MD-lactate	−*0.28*	***.046***	0.24	.09	−0.19	.18
Cerebral MD-LPR	−0.20	.15	0.17	.24	−0.17	.23
GOS-E	−0.08	.50	0.04	.72	−0.09	.45

Abbreviations: CPP, cerebral perfusion pressure; GOS-E, Extended Glasgow Outcome Scale; ICP, intracranial pressure; MD, microdialysis; LPR, lactate-/pyruvate ratio; PRx, pressure autoregulation index.

^a^ Arterial oxygenation versus MD variables (N = 51), neurophysiology (N = 57), and GOS-E (N = 67).

*P*-values in bold and italics were considered statistically significant.

### Arterial Oxygenation and Cerebral Energy Metabolic Patterns

**Figure 2.**
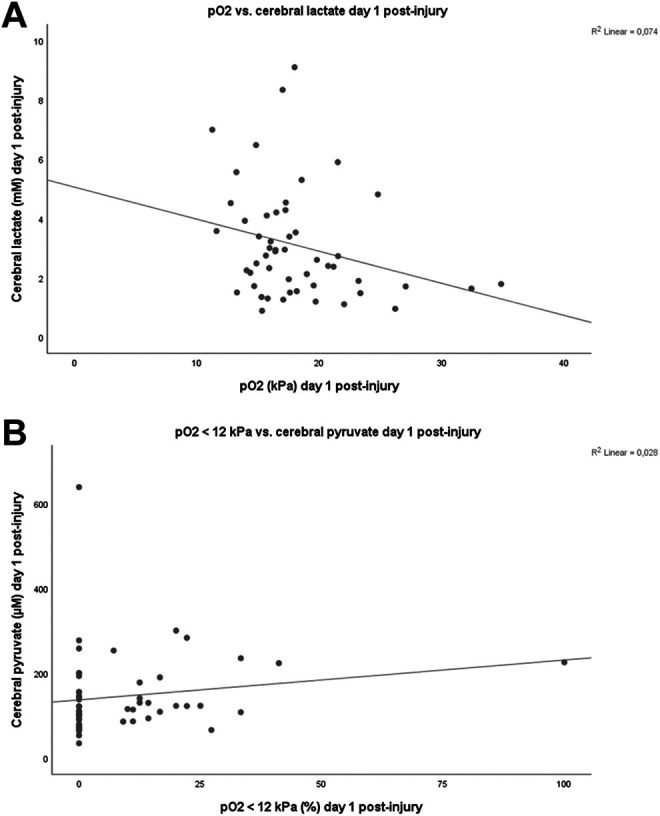
Arterial oxygenation and cerebral energy metabolism day 1 post-injury.

Higher mean arterial pO_2_ was significantly associated with lower cerebral MD-lactate (r = −0.28, *P* = .046; Figure 2A, Table 2). The mean pO_2_ did not correlate with the other energy metabolites (cerebral MD-glucose, MD-pyruvate, and MD-LPR; Table 2). The percentage of ABGs below our oxygen target 12 kPa was associated with higher cerebral MD-pyruvate (r = 0.28, *P* = .046; Figure 2B, Table 2). There was no significant correlation between the percentage of hyperoxia and the cerebral energy metabolites (Table 2). Similar correlation analyses using the Δ(Day 2 − Day 1) values did not reveal any significant associations between changes in arterial oxygen levels and cerebral energy metabolites. Exclusion of those patients treated with DC (n = 7) on the first day post-injury did not have any impact on the results.

**Figure 3.**
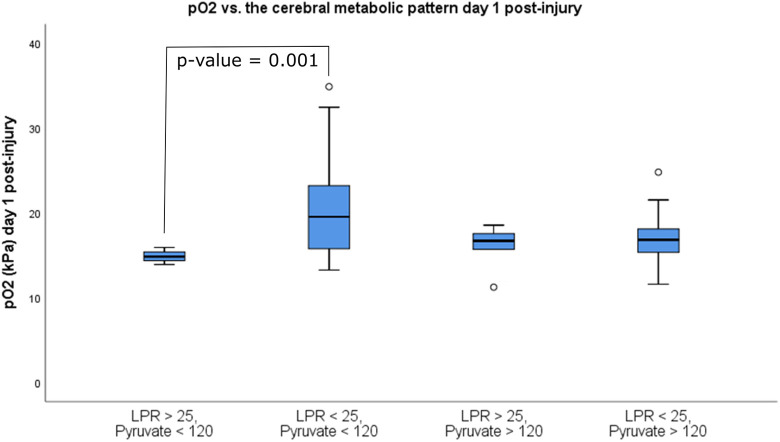
Arterial oxygenation in relation to cerebral metabolic patterns day 1 post-injury.

Patients (N = 51) were divided into 4 groups based on their metabolic pattern of mean MD and pO_2_ values for day 1 (Figure 3). The patients with a metabolic pattern of “energy metabolic disturbances and limited pyruvate supply” (cerebral MD-LPR > 25 and MD-pyruvate < 120 µM, N = 4) had significantly lower mean pO_2_ at 15 ± 1 kPa compared with those without metabolic disturbances but limited pyruvate supply (cerebral LPR < 25 and pyruvate < 120 µM, N = 21) at 20 ± 6 kPa (*P* = .001). Both of these groups had similar P/F (pO_2_/Fio_2_)-ratios (43 ± 11 vs 57 ± 16, *P* = .10), GCS M at admission (4 ± 2 vs 5 ± 1, *P* = .69), and Marshall score (3 ± 1 vs 3 ± 1, *P* = .95). Furthermore, there was no significant difference in arterial oxygen levels between those with energy metabolic disturbances with limited substrate supply (LPR > 25 and pyruvate < 120) versus those with energy metabolic disturbances with normal substrate supply (LPR > 25 and pyruvate > 120; 15 ± 1 vs 16 ± 3 kPa, *P* = .40). Exclusion of those patients treated with DC on the first day post-injury did not have any impact on the results.

Of those with a mean cerebral pyruvate below 120 µM and LPR above 25, all but one patient had a mean cerebral MD-glucose above 1 mM. None of these patients had an ICP > 20 mm Hg or CPP < 60 mm Hg. All patients had a mean Hb above 100 g/L. There was no significant difference in mean ICP, CPP, and pCO_2_ between those with low cerebral MD-pyruvate and normal versus elevated MD-LPR (data not shown).

### Arterial Oxygenation and its Relation to ICP, CPP, and Pressure Reactivity

Although the mean pO_2_ or ABG events below our oxygen target did not correlate with ICP or CPP, higher percentage of hyperoxia day 1 was significantly associated with lower ICP (r = −0.28, *P* = .04; Table 2). Furthermore, the correlation analyses of the (Δ(Day 2 − Day 1) values showed that patients who had decreased percentage of pO_2_ > 20 kPa had a significantly higher ICP (r = −0.29, *P* = .03) and a significantly lower CPP (r = 0.28, *P* = .03) from day 1 to day 2 post-injury.

**Figure 4.**
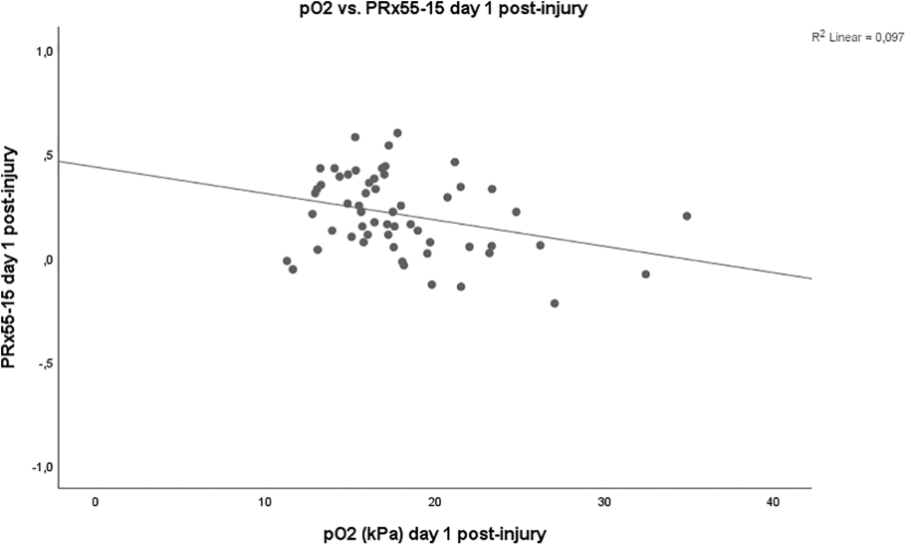
Arterial oxygenation and pressure reactivity.

In addition, higher mean pO_2_ was significantly associated with lower PRx55-15 (r = −0.30, *P* = .02) day 1 post-injury (Figure 4). Similarly, the correlation analyses of the (Δ Day 2 − Day 1) values showed that those patients who had decreased in mean pO_2_ had significantly higher PRx55-15 (r = −0.37, *P* = .005) from day 1 to day 2 post-injury. However, the association between arterial oxygenation and PRx55-15 was not significant in the later course (Spearman correlation test of mean values day 2-5 and day 6-10, respectively). Exclusion of those patients treated with DC on the first day post-injury did not have any impact on the results.

In the multiple linear regression analysis for PRx55-15 (Table 3), including age, ICP, CPP, pCO_2_ and pO_2_, higher pO_2_ was marginally associated with lower PRx55-15 (*P* = .054).

**Table 3.** Prediction of PRx55-15 Day 1 Post-Injury—A Multiple Linear Regression Analysis.^a^

Physiological Variables	β	*P* value
Age	−0.08	.60
ICP	0.22	.20
CPP	0.02	.93
pO_2_	−0.27	.054
pCO_2_	0.18	.17

Abbreviations: ANOVA, analysis of variance; CPP, cerebral perfusion pressure; ICP, intracranial pressure; PRx, pressure autoregulation index.

^a^ N = 56, *R* ^2^ = 0.19, ANOVA of the regression, *P* = .05.

## Discussion

In this retrospective study including 115 patients with severe TBI, we found that high mean arterial pO_2_ was associated with better PRx55-15 and improved oxidative cerebral energy metabolism on the first day post-injury. In TBI patients with a cerebral metabolic pattern indicative of *limited* pyruvate supply, those with preserved oxidative energy metabolism (normal cerebral MD-LPR) had significantly higher arterial oxygen (20 vs 15 kPa) than those with energy disturbances (elevated MD-LPR). This suggests that TBI patients with disturbed energy metabolism with limited cerebral MD-pyruvate may benefit from a higher pO_2_-threshold than 12 kPa in early TBI management, although prospective studies are needed to validate this.

### Arterial Oxygen and Cerebral Energy Metabolism

Cerebral ischemia and hypoxia are common secondary insults following severe TBI.^42,43^ Optimizing cerebral oxygen delivery by maintaining adequate CBF and arterial oxygen pressure has been discussed extensively the last decades.^2^ This has traditionally been achieved by CPP-oriented treatment protocols to increase CBF^44^ and maintaining arterial oxygen content sufficient by keeping Hb above 70 to 100 g/L together with normal saturation.^28,45^ More recently, NBO treatment has been suggested to compensate for cerebral ischemic hypoxia, overcome diffusion barriers from cerebral edema, and to improve mitochondrial function in TBI.^2^

The benefits of NBO are debated.^2^ Diringer et al found no global cerebral improvement in CMRO_2_ following NBO,^46^ whereas Nortje et al found increased CMRO_2_ in ischemic regions.^16^ Furthermore, many energy metabolic studies have found reductions in cerebral lactate following NBO, but no improvement in MD-LPR.^10,12–15^ However, another study found that the beneficial effect of hyperoxia depends on the concurrent cerebral energy metabolic state, as improvements in oxidative energy metabolism were only seen in cases of high cerebral MD-lactate before treatment initiation_._^17^ Similar to these previous studies,^10,12–15^ our patients who had higher mean arterial oxygen levels had lower cerebral lactate (Figure 2A) and those with higher percentage of ABGs with pO_2_ < 12 kPa had higher cerebral MD-pyruvate levels (Figure 2B), possibly as glycolytic enzymes were upregulated due to hypoxia. The correlation between arterial oxygenation and MD-LPR was modest and nonsignificant, but similar to Vilalta et al,^17^ improvements in oxidative energy metabolism from higher pO_2_ could be seen under certain circumstances in our study. Our patients with normal oxidative cerebral energy metabolism (MD-LPR below 25) with concurrently low cerebral pyruvate, indicative of *limited* substrate supply,^41,47^ had significantly higher arterial oxygen levels compared with those with *limited* substrate supply and deranged oxidative energy metabolism (Figure 3). However, there was no significant difference in P/F-ratio between the groups, indicating that pO_2_ rather than lung injury could explain this association and that TBI patients with *limited* energy substrate supply could benefit from higher oxygen levels. No patient with *limited* substrate supply had concurrent ICP above 20 mm Hg or CPP below 60 mm Hg and anemia and cerebral MD-glucose below 1 mM were uncommon. This indicates that macrovascular ischemia and arterial oxygen content were usually not the cause of the metabolic disturbances. Instead, microvascular disturbances, diffusion limitation, and hypermetabolism seem more plausible.^9^ Furthermore, it has been suggested that arterial hyperoxia could be beneficial in case of mitochondrial dysfunction, but we did not find any difference in arterial oxygenation between the patient groups with normal cerebral MD-pyruvate supply with either normal versus disturbed oxidative energy metabolic state (low/high MD-LPR). However, it is difficult to evaluate the energy metabolic effects of different oxygen levels from this analysis, since the extent of mitochondrial injury in each group is unknown and probably differ. It would be better to evaluate the effect of arterial oxygenation prospectively in future trials with a hyperoxic challenge in patients with a metabolic pattern indicative of mitochondrial failure.

### Arterial Oxygen and Cerebral Autoregulation

Cerebral autoregulation may become dysregulated in TBI and this is associated with poor outcome.^31^ The cerebral vasoresponse to various regulators such as arterial blood pressure, pCO_2_, and cerebral energy metabolism represent the general health of the vessels and intact vessel response to one of these regulators usually corresponds to intact vessel response to the other regulators.^20^ It is well known that arterial hyperoxia generates an autoregulatory reduction in CBF to maintain a normal pBtO_2_^24,25^ and this has been further elaborated in the NIC setting by, for example, evaluating the cerebral TOR to an increase in Fio_2_. Tissue oxygenation response is highly correlated with PRx,^27^ indicating that these indexes evaluate different but still common aspects of general cerebral autoregulation. In this study, we found that higher arterial oxygen levels were associated with better pressure reactivity (Tables 2 and 3). Hypoperfusion and pressure passive cerebral vessels is a well-known complication the first day following severe TBI and increased vascular tone following hyperoxia could then improve general cerebral vasoreactivity.^48,49^ This highlights that autoregulatory-oriented therapy not only includes CPP and PRx but rather is multidimensional and could be improved by optimal arterial oxygenation. However, the association between arterial oxygenation and PRx55-15 was not significant in the later acute course post-injury. It is possible that the pO_2_-variation in the later course post-injury (Figure 1) was too small to detect any difference of arterial oxygenation on PRx55-15 or temporal differences in vessel response to arterial oxygen.

### Arterial Oxygenation in Relation to Clinical Outcome

Too low and too high arterial oxygen levels have been shown to be associated with worse outcome following TBI.^50–52^ Furthermore, low pBtO_2_ has also been found to correlate with worse outcome.^4,5^ Although we did not find any correlation between higher arterial oxygen levels the first day post-injury and better clinical outcome, we did find correlated improvements in PRx55-15 and oxidative cerebral energy metabolism. Possibly, the primary brain injury and other secondary insults had greater impact, confounding any impact on functional recovery as measured using GOS-E. Alali et al^52^ found that modest arterial hyperoxia (20-25 kPa) day 1 post-injury was associated with favorable clinical outcome in a study including 417 patients, indicating that our study may be underpowered to detect a small, but significant, effect of arterial hyperoxia on outcome.

### Limitations

First, although pO_2_ and pBtO_2_ are correlated, we lacked sufficient data regarding cerebral oxygenation to evaluate the relation between pO_2_, pBtO_2_, and cerebral energy metabolism. Second, the number of patients with energy metabolic disturbances (MD-LPR > 25) was low, limiting the reliability of the analysis in Figure 3. Third, the associations between arterial oxygen levels and cerebral energy metabolism and PRx55-15 were weak, indicating that other factors could be more important. Fourth, the physiological variables were measured over different time intervals, for example, PRx55-15 (minute-by-minute), cerebral energy metabolism (every hour), and ABGs (every fourth hour). Considering the complexity of the data regarding differences in the frequency of measurements and for what time duration each measurement is relevant, we evaluated averaged values over 24 hours to determine the correlation of the total exposure of various physiological variables. Measures of energy metabolism minute-by-minute may be more time-compatible with the other physiological measures (PRx55-15/ICP and SaO_2_) and could in the future help determine the correlation between these physiological variables with higher resolution. There is also a need for prospective trials to better study the intraindividual neurophysiological response to hyperoxia. Fifth, the association between arterial oxygen levels and cerebral energy metabolism may be confounded by other important variables such as injury severity. However, we found no association between thoracic/extracranial injuries and arterial oxygen content indices.

## Conclusions

Higher mean arterial oxygen levels the first day post-injury in severe TBI patients was associated with better PRx55-15 and improved oxidative cerebral energy metabolism. Particularly, patients with a cerebral metabolic profile indicative of *limited* energy substrate supply could benefit from higher arterial oxygen levels in the early phase of TBI. Furthermore, arterial oxygen may be used to optimize cerebral vasoreactivity and hence used in an integrated autoregulatory-oriented treatment regime. Future prospective studies are needed to determine whether there is a causal relationship of arterial hyperoxia in relation to autoregulation, cerebral energy metabolism, and clinical outcome.
